# No size fits all – a qualitative study of factors that enable adaptive capacity in diverse hospital teams

**DOI:** 10.3389/fpsyg.2023.1142286

**Published:** 2023-07-06

**Authors:** Birte Fagerdal, Hilda Bø Lyng, Veslemøy Guise, Janet E. Anderson, Siri Wiig

**Affiliations:** ^1^Faculty of Health Sciences, SHARE – Centre for Resilience in Healthcare, University of Stavanger, Stavanger, Norway; ^2^Department of Anesthesiology and Perioperative Medicine, Monash University, Melbourne, VIC, Australia

**Keywords:** resilience, resilient healthcare, adaptive capacity, teams, teamwork, quality

## Abstract

**Introduction:**

Resilient healthcare research studies how healthcare systems and stakeholders adapt and cope with challenges and changes to enable high quality care. By examining how performance emerges in everyday work in different healthcare settings, the research seeks to receive knowledge of the enablers for adaptive capacity. Hospitals are defined as complex organizations with a large number of actors collaborating on increasingly complexity tasks. Consequently, most of today’s work in hospitals is team based. The study aims to explore and describe what kind of team factors enable adaptive capacity in hospital teams.

**Methods:**

The article reports from a multiple embedded case study in two Norwegian hospitals. A case was defined as one hospital containing four different types of teams in a hospital setting. Data collection used triangulation of observation (115 h) and interviews (30), followed by a combined deductive and inductive analysis of the material.

**Results:**

The study identified four main themes of team related factors for enabling adaptive capacity; (1) technology and tools, (2) roles, procedures, and organization of work, (3) competence, experience, knowledge, and learning, (4) team culture and relations.

**Discussion:**

Investigating adaptive capacity in four different types of teams allowed for consideration of a range of team types within healthcare and how the team factors vary within and across these teams. All of the four identified team factors are of importance in enabling adaptive capacity, the various attributes of the respective team types prompt differences in the significance of the different factors and indicates that different types of teams could need diverse types of training, structural and relational emphasis in team composition, leadership, and non-technical skills in order to optimize everyday functionality and adaptive capacity.

## 1. Introduction

Resilience in healthcare (RiH) can be defined as ‘the capacity to adapt to challenges and changes at different system levels, to maintain high-quality care’ p. 6 (Wiig et al., 2020). The research within this field seeks to understand how healthcare organizations cope with the dynamic, variable, and demanding environment in which they operate based on insights from complexity and system theory and provides an alternative complementary perspective of learning from and understanding how, most of the time, work is safe (Ellis et al., 2019; Anderson et al., 2020a; Iflaifel et al., 2020; Ignatowicz et al., 2023). By examining how performance emerges in everyday work in different healthcare settings, RIH research seeks to develop knowledge of the enablers for adaptive capacity in everyday work, the focus is on how systemic and organizational processes can support adaptations, rather than on how individuals are resilient handling stressful events. Adaptations occur constantly in healthcare work in response to disruptions (positive and negative), shocks or crises, and are essential for maintaining control and the ability to function (Blanchet et al., 2017; Lyng et al., 2022). This ability for adaptive capacity in healthcare can be conceptualized as constituting “adaptations based on reframing, aligning, coping and innovating, in response to external and internal demands from different organizational levels, in order to ensure quality of care.” p. 7 (Lyng et al., 2022), and may be anticipated or unanticipated, short, or long term, and, occasional or regular (Lyng et al., 2022).

Hospitals are defined as complex organizations with high task demands and a large number of actors collaborating across time and space to deliver safe healthcare. The tasks and number of interactions are high and variability in performance frequently occurs (Braithwaite et al., 2015). This inherent complexity requires healthcare professionals from multiple disciplines to co-ordinate their actions in teams. Consequently, most of the work currently being done in hospitals is team based. Teams are a means of organizing work so that individuals can accomplish more than they can on their own and to maintain operations 24 h a day (Bell et al., 2018). Hospitals as well as other healthcare organizations depend on teams to successfully undertake increasingly intricate tasks (Flin and Maran, 2004).

### 1.1. Teamwork in hospitals

A common conceptualization of teamwork is “two or more individuals with specified roles interacting adaptively, interdependently, and dynamically toward a common or valued goal” p. 559 (Salas et al., 2005). Teamwork includes skills in communication, team leadership, anticipation, feedback, and support, along with each team members ability to understanding their role and responsibility (Salas et al., 2005). The quality of teamwork is closely related to quality and patient safety in treatment and care (Salas et al., 2005; Rosen et al., 2018). Team training is important for improved efficiency in inter-professional teamwork within hospitals (Ballangrud et al., 2017). A vast number of overview articles and studies on teamwork and team training in healthcare have been published over the past decades (Hughes et al., 2016; Chen et al., 2019; Gross et al., 2019; Gregory et al., 2021). A significant amount of the literature has focused on Crew Resource Management (CRM) and TeamSTEPPS in limited clinical fields of practice where the results mainly have focused on participants reactions and the degree of learning achieved (Ballangrud and Husebø, 2021). Furthermore, the human factors discipline has focused on improving the quality and safety of care by focusing on teamwork with the development and refinement of the System Engineering Initiative for Patient Safety model (SEIPS 3.0) (Carayon et al., 2020). Further research on teams is needed to identify important factors for long term sustainability of team competencies (Ballangrud and Husebø, 2021). It is well recognized that teamwork in hospitals comes with multiple challenges (Weller et al., 2014; Anderson and Reedy, 2021). Team members may come from different professional backgrounds with different training, knowledge, and attitudes. They often, work shifts and are located in different spaces across the hospital. Teams are diverse in structure and purpose (WHO, 2009; Anderson et al., 2020a) and a new approach to understanding teamwork is therefore needed to understand the commonalities, differences, challenges, and success factors of different types of teams. Recent literature on resilience in teams, identifies four types of teams that are common in the hospital setting (Anderson et al., 2020a); (1) Structural teams which are co-located, uni/multi professional, and feature prolonged teamworking; (2) Hybrid teams which have some permanent and some rotating staff, and feature planned teamwork; (3) Responsive teams, which respond to acute and unplanned episodes of teamwork, usually organized as mobile teams; and, (4) Coordinating teams in which planned episodes of teamwork integrate representatives from multiple teams, usually spanning different hospital units (see Table 1). Although it is clear that teamwork is fundamental to work in hospitals we need more knowledge on how team, organizational and system factors combine to influence team performance (Anderson et al., 2020a) and adaptive team capacity specifically. The rationale for this study is to increase our knowledge on how resilience is enabled in healthcare systems by studying adaptive capacity in different forms of hospital teams. As such we aim to identify what type of team factors that are of importance in enabling resilience.

**Table 1:** Team descriptions.

Team type	Organizational Context/structure	Demands/processes	Misalignments	Location
Structural	Ward based, nurses and assistants working together in small units 3–4 persons. Co-located	Receive patients 24/7 Unpredictable workday	Lack of staff, competence Peak situations	Orthopaedic/surgical bed ward Neurological bed ward
Hybrid	Permanent staff of nurses, rotating medical staff, co-located	Receive acute patients 24/7 Unpredictable workday Rapid workflow changes	Lack of staff, competence Peak situations	Emergency department Short stay acute unit
Responsive	Acute teams responding to incidents of cerebral infarction. Multi professional. Short episodes of teamwork	Respond to suspected cerebral infarction Routine work	Workflow changes due to patient situations	Members from differerent departments
Coordinating	Meeting of ward managers allocating patient to even out demand and capacity in the hospital. Their work span hospital units	Solve overall capacity in the hospital Complex organization	Inconsistant patient numbers	Members (Ward managers) from different departments

### 1.2. Aim and research question

The aim of this study was to increase our understanding of adaptive capacity in four different types of hospital teams by exploring the temporal and dynamic features of teamwork and the contextual influences within which they operate. More specifically this study investigates how team factors (e.g., competence, team culture, procedures) relate to teams’ adaptive capacity in four different team types in two hospitals. The following research question guided the study: what kind of team factors enable and hinder adaptive capacity in teams, and how do these factors affect adaptive capacity?

## 2. Methods

### 2.1. Design and setting

A qualitative case study methodology was used to explore team factors and how they enable adaptive capacity in hospital teams. Qualitative research describes, interpret, and generate theories about social interactions and individual experiences as they occur in natural, rather than experimental, situations (O’Brien et al., 2014; Busetto et al., 2020). The study was designed as a multiple embedded case study conducted in two Norwegian hospitals (Yin, 2014). A case was defined as one hospital containing four different types of teams.

### 2.2. Recruitment and study context

In line with the study protocol (Anderson et al., 2020a) the two hospitals were selected and recruited based on their size and teaching position. Hospital 1 is a large teaching hospital with both national and regional responsibilities in addition to local functions and Hospital 2 is a middle-sized local hospital in the Norwegian healthcare context. Both the hospitals are situated in the same health region and collaborate to provide local functions. In the Norwegian health system responsibility for healthcare service provision is divided between local municipalities and four regional health authorities. The municipalities are responsible for primary care services for their citizens, including nursing homes, homecare, general practitioners, and rehabilitation services, while the four regional health authorities are responsible for the specialized healthcare services, including the governance of hospitals.

To gain initial access to the recruited hospitals we contacted their respective research departments, and researcher BF used her professional network to contact key personnel in the departments, enabling us to perform data collection during the Covid-19 pandemic. After receiving permission and access to carry out the study in both hospitals, we collaborated with the hospitals to identify and locate the four different team types in each of the hospitals; structural, hybrid, responsive and coordinating (see Table 1). The leaders of the identified teams were then approached directly. They were provided with detailed information about the study and given time to consider whether to participate. A total of four teams were recruited from each hospital (total of eight teams) to participate in observations of their work practice and in interviews. The compositions of the teams were similar in both hospitals, where members of the structural and hybrid teams were mostly nurses and nursing assistants alongside a smaller number of physicians, the numbers of which could vary from shift to shift. The responsive teams consisted of a permanent set of members from diverse healthcare professions. The coordinating teams consisted of ward managers from the different bed wards in each hospital. Due to the difference in size and number of wards in the two hospitals, Hospital 1 had a much larger coordinating team than what was the case in Hospital 2. During the observation we recruited participants for interviews. Researcher BF made appointments with the participants and the interviews were undertaken after the observation period was completed. Three to four team members in each team and one leader in each team were interviewed. A total of 30 interviews were conducted (see Table 2).

**Table 2:** Overview of data collection methods and data material according to team types and case sites.

Hospital 1			Hospital 2		
Team	Observation	Interview	Team	Observation	Interview
Structural	29 h	3	Structural	29 h	4
Hybrid	14 h	4	Hybrid	27 h	5
Responsive	(30 h)/ 3 h	4	Responsive	1 h	3
Coordinating	6 h	3	Coordinating	6 h	4
Sum	52 h	14	Sum	63 h	16

### 2.3. Data collection

We collected data through observation, interviews, and document analysis. The data were collected between December 2020 and June 2021. Researchers BF and HBL conducted the observations of all the teams using an observation guide which was subsequently used to structure the writing of the field notes. Both researchers wrote their own field notes for each of the teams. The guide was developed in line with central concepts from the resilience literature, and essential features of hospital teams. This prompted the researchers to capture key aspects of work as done (Anderson et al., 2016). The researchers looked for types of demands from the different levels in the organizations, capacities of the team to meet demands and types of adaptations that were performed. As teams were different in how they worked together, the length and timing of observations had to align with that. For the structural and hybrid teams the researchers shadowed one or more team members for an evening shift and the following dayshift. For the responsive teams the researchers shadowed the team members during their shift and followed them when they responded to acute alarms. The coordinating team met 10 to 15 min for a daily planned meeting. The two researchers observed the coordinating team meetings for a two-week period. As one of the coordinating teams held the meeting digitally due to the Covid 19 pandemic, the researchers attended this meeting digitally together with the rest of the team. The observations across all eight teams resulted in a total of 115 h of observation.

We used a semi structured interview guide based on content from the Concepts for Applying Resilience Engineering (CARE) model, i.e., demand, capacity, misalignments, and adaptations (Anderson et al., 2016). And furthermore, the four potentials of resilience; monitoring, anticipating, responding, and learning (Hollnagel, 2018). By conducting the interviews post observation, we were also able to ask about situations that we had observed and discuss them with participants to elaborate the adaptations they made in the course of their work. Researcher BF conducted all the interviews. Most of the interviews were held face to face at the respective participant’s workplace, but some were held digitally due to the Covid-19 pandemic and consequent social distancing regulations. The length of the interviews varied from 40 to 90 min, with a median length of 60 min. All interviews were audio recorded and transcribed verbatim by researcher BF. The data material, including the transcribed interviews and observation notes totaled 430 pages (see Table 2).

### 2.4. Analysis

Observation notes and interviews for each team were transcribed grouped together to simplify the analysis work. The analysis was performed with a combined deductive and inductive approach (Elo and Kyngäs, 2008). We used the CARe model (Anderson et al., 2020b) as a framework to facilitate the deductive analysis. First, observation notes and interview transcripts were read through by the individual members of the research team to get a sense of the whole material and to select the units for analysis. To organize the data, we developed a categorization matrix based on the CARE model’s key concepts of demand, capacity, misalignments, and adaptations. We further used NVIVO software to deductively select and code units for analysis according to three of the four main categories of the matrix: ‘capacities’, ‘misalignments’, and ‘adaptations’. The capacities category was renamed ‘team factors’ and represent factors that have a positive influence on the ability to adapt. All data were additionally coded according to team type and hospital, which allowed for cross-team and cross case analysis. This resulted in a substantial number of different activities for each of the categories. The material in the three deductive categories was further analyzed following the principles of inductive content analysis (Elo and Kyngäs, 2008) (see Table 3). All the data within the categories were inductively reviewed and recoded which were then further developed into themes across teams. There were differences between the teams, but in our cross-team- and cross case analysis we found overarching themes that matched all eight teams and their variances and nuances in how team factors influence adaptive capacity. The overarching themes enabled us to identify patterns, similarities and differences across both teams and hospitals, which deepened our understanding of team adaptive capacity. We transferred the themes into tables and developed a heatmap to visualize the differences in the various teams (see Table 4). Table 4 provides an illustration of differences between the different teams and hospitals based on instances of team factors that were noted in the qualitative data material. The number of team factor instances across teams and hospitals are represented with various colors ranging from green (few) to red (many).

**Table 3:** Deductive categories analyzed following the principles of inductive content analysis.

Category: team factors			
Quote	Category	Code	Themes
*“While reading, they have a printed list from the electronical patient software that they use to make notes on. In addition, this list serves as a support to know the most important matters about the other patients for whom they are not responsible but may need to be able to help during their shift.”*Observation notes Structural team (1)	The use of lists with key features enables swift and correct assistance	Tools for information and preparing	Technology and tools
*“In the nurses’ workstation the team members always keep an eye on the screen of incoming patients in order to be prepared.”*Observation notes Hybrid team (2)	Available software improve preparedness	Sufficient equipment	
*“We have a form with dosage of anticoagulant by weight hanging on the wall. We use that instead of calculating it ourselves.”*Physician Responsive team (2)	Predefined dosages hinder miscalculations	Written tools on display for easy access	
*“Yes, we’ve been doing it for a few years now, had these meetings. We’ve had them longer, but there’s no need to have them that long. We’ve made some changes to the “structure over time. For example, when we’ve got the patient overview software, we don’t have to say all the numbers, because everybody can see them.”*Head nurse, Coordinating team (2)	Software enables more efficient meetings	Tools for information provides for more efficient meetings	
*“We have divided them into groups, yard 2 and yard 1, after 3 months they switch, so that they get variety. Sometimes you have to work in yard 1 even if you belong to yard 2 due to illness or that there were only new employees staffed there, but mostly so, yes… So today they switch groups on one side, and that’s how it works.”*Leader structural team (2)	Team belonging, but system for changes due to illness	Work routines to even out workload	Roles, procedures, and organization of work
*“The ward also has a nurse working an intermediate shift from 11 am. to 7 pm. This role does not have defined tasks but helps where needed. Often the intermediate shift takes care of new patients who are admitted to the ward.”*Observation notes, Hybrid team (2)	Planning for peak hours	New roles to even out peak hours	
*“The procedure document clearly describes the physician as the leader of the team. It is also clearly described what the team leaders’ focus and tasks should be.”*Observation notes Responsive team (1)	Clear description of work tasks	Procedure for division of responsibility	
*“The meetings follow a firm structure where everyone present gets their turn to speak. Before the meeting all the wards have filled in the day’s patient numbers in the software programme they use. The meeting is led by a placement coordinator.”*Observation notes Coordinating team (1)	Clear roles and firm management of meetings	Roles for structure enables efficient meetings	
*“We try to do tutoring and training regularly. We arrange lesson in medical topics that are relevant to us. We train in the use of medical equipment regularly. And we try to train new nurses…And also through staff appraisal and such we assess whether there is a need for any training.”*Leader Structural team (2)	Competence development in the team	Learning activities enhance competence	Competence, experience, knowledge, and learning
*“Nurses in the emergency department are organized according to competence levels. All new employees, regardless of which department they have worked in before, start at competence level 1 (after an introduction period), after a minimum of one year they can move up to the next competence level, before ending up at competence level 3 after a variable period. Different situations and roles on the different shifts require different competence levels. Cardiac arrest and actilyse (thrombolysis) require competence level 1. Red alarms and shift leader require competence level 3.”*Observation notes, Hybrid team (2)	Competence levels secures correct and sufficient competence present on all shifts	Division of competence to assure quality	
*“The staff talk about how experienced staff members contribute to the procedure being performed more rapidly. They praise the experience of the thrombolysis nurses who will contribute more if the neurologist is less experienced.”*Observation notes Responsive team (1)	Certain roles and teams require experience	Experience contributes to competence and safety	
*“I guess it depend on who’s at the meetings. Some are easier… In other words, for some it is easier to find a solution than for others. It depends a on the participants, how experienced they are amongst other things. Because this is a team that is not made up of the same members every day.”*Head nurse Coordinating team (1)	Experience enables the members with more options for solving problems	Experience contributes to a wider range of solutions	
*“Everyone is good at asking: “Can I do something for you?” If one of us is very busy, the others chip in to help, so that no one is sitting around doing nothing, while others are working their ass of.”*Nurse 3 Structural team (1)	Well acquainted team members develop a culture for helping each other	Helping culture enables overall capacity	Team culture and relations
*“We cooperate closely, we communicate a lot during the shift about things and situations we need to look out for and if we need to watch someone’s patients for a period, when they are occupied with another situation. So, we collaborate really well, and are very understanding of each other’s needs.”*Nurse 1 Hybrid team (1)	Close communication between team members and understanding each other’s needs	Collaboration and understanding of each other’s roles	
*Yes, basically it is the case that the team, seldom, or you could say never, meet in the same constellation since there are numerous departments and sections involved and each of them has a lot of employees. So, in the context of brain stroke, good team collaboration actually means that we save an incredible amount of time. For us, the whole stroke collaboration is built on us spending as little time as possible until the patient gets the right treatment so everyone has to know their procedures, they have to know exactly what to do, and they have to know all the things they shouldn’t do to not delay the treatment.”*Leader, Responsive team (1)	Team culture of mutual understanding of their roles and tasks	Focus on roles for good collaboration	
*“I felt like you got some insight into what the other wards were doing. Also, you felt, what I think is most important, is that you felt a little bit of that responsibility. You just have to deal with it and, if some wards were really busy or, you had to deal with each other, and I think that’s very healthy, that you shouldn’t just think of yourself and your ward in a way.”*Head nurse, Coordinating team (1)	Development of a culture for helping each other	Feeling responsible for others situation enables overall capacity	

**Table 4:** Heatmap of team factors (Themes).

	Coordinating H2	Coordinating H1	Hybrid H2	Hybrid H1	Responsive H2	Responsive H1	Structural H2	Structural H1
1: Technology and tools	6	4	3	9	7	18	4	1
2: Team culture, relations	27	31	30	37	3	20	40	27
3: Roles, prosedures, and organizations of work	38	32	41	18	21	51	23	21
4: Competence, experience, knowledge, and learning	3	0	16	12	8	27	16	10

## 3. Results

The results of how team factors influenced adaptive capacity in the eight teams at the two hospitals are presented team-wise and structured according to the following four main themes: (1) Technology and tools, (2) Roles, procedures, and organization of work, (3) Competence, experience, and learning, and (4) Team culture and relations. See Table 3 for an overview of the themes with quotes from the interviews or observation notes.

### 3.1. Structural teams

The structural teams were ward-based teams that consisted of nurses and nursing’ assistants working together on a permanent basis. They worked together in small units of 3–4 persons on each shift. Typical misalignments for the teams were the unpredictable workday, lack of staff, competence (e.g., staff on sick leave with no proficient substitute available) and challenging peak situations (sudden high flow of incoming patients).

#### 3.1.1. Technology and tools

An important enabling factor for the structural teams was the availability of technology and tools (e.g., computers, software’s, mobile devices, electronic equipment for monitoring patients), and how the organization supported the teams by making these tools available, alongside the physical workspaces that corresponded with their needs. The structural team members in both hospitals carried a printed list containing names, diagnoses, and treatment plan for all patients in their pockets. The list was updated in the software and printed out at the start of each shift. The list not only helped the team members to easily assist each other in the treatment of patients, but also to monitor the overall status of the ward. In hospital 2 everyone also had their own mobile device. With this device they signed on to the care of their patients in a program that enabled other partners at the hospital to promptly contact them about their patients.

#### 3.1.2. Roles, procedures, and organization of work

Organization of work, the structure of the different shifts, clear role descriptions and procedures for work tasks were factors that provided the teams confidence to undertake their daily work. Although the teams had a plan, they were always prepared for it to change. Adaptive capacity was enabled by planning how to support each other, and by being mentally ready for changes to happen as part of a normal workday. Moreover, they prepared for the absence of team members who held additional roles outside the team (e.g., those who were part of a responsive team), for example by avoiding allocating responsibility for the most severely ill patients to them.

The results show how the organization of work took normal peak hours of the day into account by providing extra floating staff for these periods. The extra resources served the teams on the ward and completed requested tasks to reduce the misalignments of demand and capacities.

A key factor for supporting adaptive capacity related to how the team members continuously updated each other within the team during the shift. Updates enabled the organization of relevant assistance for patients and preparations for emergencies, for instance in the case of a deteriorating patient. It was also important for the team members to know who was available to help out, and scheduled team huddles helped bring the team members up to speed.

#### 3.1.3. Competence, experience, knowledge, and learning

Competence was vital for the structural teams’ ability to adapt. Several daily work tasks required high professional competence, such as monitoring acutely ill patients, or patients newly transferred from the intensive care unit (ICU). Replacing competent team members with less competent members led to a redistribution of tasks, and increased responsibility for others with more competence. The team members’ experience was also important for adaptive capacity. When faced with challenges, experienced team members brought a sense of safety to the teams, and they supported new team members by providing advice. For example, knowing what to prioritize in peak situations was something new nurses found challenging to do as it has to be learned through working with more experienced colleagues. Competence development such as tutoring, training and simulation was offered to structural teams, but often competed with the daily chores.

#### 3.1.4. Team culture and relations

The structural team members supported each other in carrying out their work. A culture of helping each other was fundamental for their adaptive capacity, for example by making sure that everyone got a break during the shift or helping with tasks if one of the members was struggling. Findings show that team members who work regularly together on weekend shifts became very familiar with each other and thus developed their own structures for the division of responsibility and support for each other. Team members talked about knowing each other personally and professionally. The division of responsibility on a shift was easier if they knew each other’s preferences, strengths, and weaknesses. It was also easier to ask for help, or admit that they did not know, or were uncertain about something. They talked about how team members reluctant to help others, were counterproductive for the team’s adaptive capacity.

### 3.2. Hybrid teams

The hybrid teams had a permanent staff of nurses and a rotating staff of physicians. The two teams observed in this study were situated on short stay units receiving patients with a wide range of diagnoses who were admitted 24 h a day, 7 days a week. Typical misalignments for these teams were lack of staff, peak situations with high inflow of patients, erroneous triaging of incoming patients, and rapid workflow changes where the team members quickly needed to respond.

#### 3.2.1. Technology and tools

The hybrid teams in both hospitals worked in well-equipped wards with premises well-suited for their work, with open spaces and short distances enhancing the prospect of monitoring and assisting colleagues when needed. In hospital 2 they used a software program that indicates incoming patients and their status to monitor, plan and prepare for admissions.

#### 3.2.2. Roles, procedures, and organization of work

Receiving acute admission patients with a range of diagnoses, meant that the team needed clear procedures and role descriptions to structure their work. Patients were distributed within the team according to capacity, and the team members were prepared for unpredictable workdays. As in the structural teams, the hybrid teams were supported with extra floating staff to reduce workload and avoid high pressure situations during known peak hours of the day. In hospital 1 both nurses and physicians were physically located together, at the same work desk, and thus worked closely to improve information flow and communication.

In hospital 2, all patients were triaged into red, yellow, and green by ambulance personnel prior to admission. However, the coding of patients was often found to be misleading, leading to a need for the team members to reallocate resources and take on extra work to ensure quality in care in such situations.

Some hybrid team members held additional roles outside their team. Stroke alarm and cardiac arrest alarms were assigned to both nurses and physicians. In hospital 2 certain tasks and responsibilities came with the requirement of a certain level of competence, with three specified competence levels for the nursing group (1, 2, 3), and two for the physician group (1, 2).

The shift leader maintained an overview of the ward, and planned the allocation of incoming patients, and the transfer of patients to bed wards. By having an overview of the total situation, the shift leader could handle high pressure situations by reallocating resources to where they were needed. This supported the overall adaptive capacity of the team.

#### 3.2.3. Competence, experience, knowledge, and learning

The distribution of roles and responsibilities among team members reflected the competence levels of both nurses and physicians. This was of high importance especially in hospital 2, due to the many roles and competence requirements the team had to cover on a shift (e.g., shift leader, stroke alarm respondent, and cardiac arrest alarm respondent). Both hybrid teams received patients with a large range of symptoms and the teams’ task was to decide on a diagnosis and quickly start treatment. The range of possible treatments made it difficult for all team members to be familiar with all of them, so to a large degree they relied on written procedures. The less experienced the team members were, the more they relied on procedures. Several of the procedures and the different equipment in use in both hospitals required competence to operate, as well as recertification on a regular basis. This led the team to regularly undergo retraining. Both teams also conducted simulation training on a frequent basis to learn and maintain skills in undertaking critical procedures, and to handle and adapt to situations under stress.

#### 3.2.4. Team culture and relations

Both the hybrid teams were characterized by a supportive culture, where they tried having lunchbreaks together, or offered help to colleagues. In particular, the leaders in hospital 1 had put major effort into reducing the hierarchy between nurses and physicians in the team. The leaders insisted that the team members should be situated next to each other during their workday to enable information transfer and collaborative decision making. However, for the rotating physicians, familiarizing themselves with this structure was a challenge. Similar to the structural teams, the hybrid team members talked warmly of each other and found weekend shifts to be especially useful for building relationships with colleagues, as members worked closely together and developed their own routines and culture during these shifts. To enable adaptive capacity, the team members depended on helping each other to even out the workload by taking on extra work and changing the responsibility for tasks when needed.

### 3.3. Responsive teams

The responsive teams in our dataset were teams who responded to suspected incidents of cerebral infarction. The teams were multi professional, consisting of members from different professions and departments. The responsive teams only met and worked together during short, unplanned, and acute episodes. Typical misalignments for the teams were workflow deviations (e.g., several patients arriving simultaneously), personnel (e.g., insecure, less trained) and patient needs (e.g., disoriented, nauseated, non-native speaker). The teams regularly experienced that the patients were not in fact suffering a stroke, and that while they were focused on a rapid diagnosis of stroke other factors might be missed. This could lead to disagreements within the team, where some team members wanted to proceed with the stroke procedure, whilst the physician as the team leader wanted further investigations to avoid missing other important conditions.

#### 3.3.1. Technology and tools

The responsive teams relied heavily on technical aids that enabled them to perform promptly. All members carried a calling device, and they could monitor incoming patients using the hospital computer system. The physicians especially aimed to make use of this tool by having one eye on the screen whilst attending to other patients. This enabled preparation by reading the patient’s journal notes before the patient arrived. In both hospitals the computed tomography scan (CT) machine was located next to the emergency room (ER) for quick access, though in hospital 2 they had to use a more precise CT machine on another floor when the patient’s symptoms were uncertain, leading to a delay of diagnosis. However, the team adapted by performing some of the examinations during transportation to reduce the delay. Furthermore, the team had a medication bag including all necessary medication and equipment. To enable speedy work processes, the bag also included predefined medication dosages for quick administration of drugs and to eliminate the risk of miscalculations.

#### 3.3.2. Roles, procedures, and organization of work

The responsive teams had a very clear division of roles and organization of work. Everyone had their specific role with assigned tasks and followed a set procedure in order for the diagnostic process to proceed quickly. The stroke procedure was designed for everything to happen rapidly in order to start treatment as soon as possible for a better patient outcome. The clear procedure also enabled them to adapt when the patient’s condition changed or there was disruption of personnel.

#### 3.3.3. Competence, experience, knowledge, and learning

The responsive teams had to make rapid and often life critical decisions to diagnose and activate stroke treatment as appropriate. These teams thus depended on the highly competent team members making the correct decisions and performing the stroke procedure quickly. To enable adaptive capacity, the team used simulation-based training for developing individual skills, ensuring a clear role understanding, and rehearsing the stroke procedure. However, since the team members did not know each other well, they emphasized the importance of training non-technical skills like communication and team management in the simulation sessions. These skills were seen as crucial for adaptive capacity in the responsive teams. During the simulation training the facilitator offered time for reflection. The lack of regular spaces to talk about their work, implied missing potential important learning points. To compensate some of the members talked about incidents with their leader or colleague when returning to their workplace.

#### 3.3.4. Team culture and relations

Since the responsive teams did not work together on a regular basis, there were limited opportunities to develop strong relationships between the team members. In hospital 1, the team had a different composition every day, and only a few of the members knew each other. In hospital 2, the team members generally knew each other from previous stroke episodes. However, the episodes of teamwork were short and characterized by an acute atmosphere, leaving little time to get well acquainted. Furthermore, they were reliant on a team culture of mutual understanding of their roles and tasks. Knowing each other enabled adaptations from set procedures. Experienced team members were more confident in doing adaptations.

### 3.4. Coordinating teams

The coordinating teams met to allocate patients to available beds to avoid bottlenecks in peak periods and even out the total demand and capacity of the two respective hospitals. The teams consisted of all bed ward managers in each hospital. They had daily 10-min meetings during the day shift to allocate patients and resources. Like in the responsive team, the coordinating team members did not work together on a regular basis, and only met for short episodes. The major misalignments of the teams were inconsistent patient numbers and a complex hospital organization where decisions about evening out demand and capacity were made across time and place. For example, the ward managers could agree on moving patients, but they needed approval from the chief physician who could disagree, leaving them to work out another solution. Also, a mismatch in conceptualizing bed capacity across wards caused challenges for team decision making. In particular, it was difficult for the members to accept/agree that some wards needed to have free buffer beds reserved for acute patient demand, for instance for Covid-19 patients, whilst other wards had to take on extra patients and overbook their capacity. This often led to discussions and disagreements within the coordinating teams. The different cultures the members represented also became visible with some team members often offering to help, while others were reluctant. The team leaders said that this was often the same members who were either positive or negative. There were few opportunities for reflecting on and learning from previous experience, and little sharing of learning.

#### 3.4.1. Technology and tools

To get an overview of the total bed capacity status at the hospital the teams needed information decision aids and tools. As mentioned, in hospital 1 the coordinating team members took advantage of software to report bed status prior to the meeting. A lot of effort and work had been done to update the computer software system to deliver the correct information about current bed status. Hospital 2 used an online tool that showed the current bed status of every ward. However, due to the rapidly changing nature of the wards’ bed status, both the coordinating teams needed additional information from the team members to make informed decisions on the reallocation of patients and beds across the hospitals.

#### 3.4.2. Roles, procedures, and organization of work

Procedures and organization of work were important in both coordinating teams. The team in hospital 1 needed more structure because it was a larger team than in the smaller hospital 2. In hospital 1 the team members each reported the status of the current capacity on their ward via a webpage prior to the meeting. The coordination meeting was led by a coordinator from the emergency department (ED) and followed a specific agenda. In hospital 2 the meeting had no firm agenda, although the objective of the meeting was similar to that in hospital 1. In hospital 2 the team had a procedure which they roughly followed, but they usually agreed on the required actions to close the bed capacity gap. The team in hospital 2 consisted of significantly fewer members than in hospital 1, and they all knew each other well, although replacements occurred. In hospital 2, in situations when the overall bed capacity was ok, and no actions were needed, the team members used this meeting to raise other challenges and supported each other in difficult decisions.

When the overall bed capacity situation was very uncertain, the teams in both hospitals could either agree to wait and see whether the situation would resolve itself without intervention or arrange for a second meeting later in the day to reassess the situation.

#### 3.4.3. Competence, experience, knowledge, and learning

The coordinating teams used competence and experience in their decision making. The team members often used their prior experience when reallocating patients or negotiating for beds among themselves, knowing what to prepare for and expect in a situation. Based on their experience they could often foresee how a situation might develop, and as such, the appropriate alternatives for solving challenges. The more experienced leaders enacted a more independent voice in the team meetings and were listened to more by others.

#### 3.4.4. Team culture and relations

Team culture and relations differed in the two observed coordinating teams. In both teams, the team culture affected their capacity to adapt to challenges and changes. Hospital 1 had a large coordinating team with a formal atmosphere and limited to no time for informal conversations. Most of the members were not well acquainted and the meeting had a very strict agenda leaving little time to get to know each other. The leader systematically worked to build a culture where the team members would feel responsibility across wards and create a supportive culture and relations between wards to better adapt and improve the overall situation of the hospital’s bed capacity. In hospital 2, team members knew each other well, and used each other for problem solving, and to give or receive advice. When the overall capacity situation of the hospitals was good, the teams functioned well.

## 4. Discussion

In this study we have explored the role of team factors for adaptive capacity in four different types of hospital teams (structural, hybrid, responsive and coordinating) in two hospitals. We found that the main team related factors of importance for enabling adaptive capacity were (1) technology and tools available to the teams; (2) clarity and description of roles, procedures, and the teams’ organization of work; (3) the teams’ competence, experience, and knowledge which also enabled sound learning processes; and (4) the team culture and relations among the members. In the following we discuss our results in relation to previous research and suggest future research and implications for practice.

**Figure 1:**
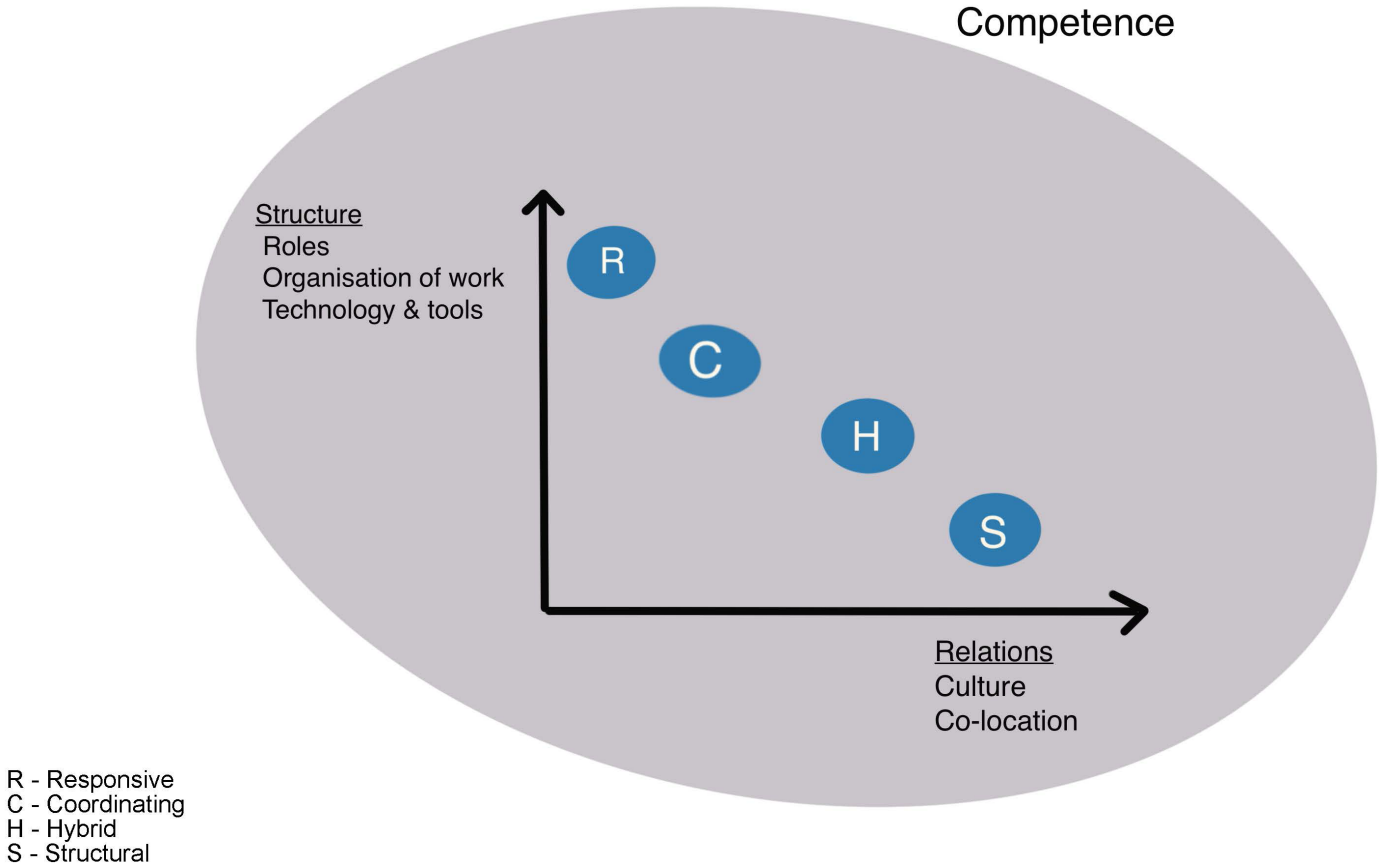
Team and factors relevance.

Our results are in line with previous research on teamwork and advances the literature on teams (Burke et al., 2006a; Rosen et al., 2011; Gittell et al., 2013; Maynard et al., 2015; Christian et al., 2017; Anderson and Reedy, 2021; Sanford et al., 2022). Our study focused adaptive capacity in four different types of teams, enabling deeper understanding of how the team factors vary within and across these teams (Anderson et al., 2020a), and how contextual factors might affect teams and adaptive capacities within teams (Schmutz et al., 2019). Although all of the four team factors identified in this study are of importance in enabling adaptive capacity, some are more important than others, as shown in Figure 1.

As Figure 1 shows, competence (experience, knowledge, and learning) is vital for adaptive capacity in all teams and is illustrated in the figure as a ring across the teams. Team performance emerges from individual cognitive and behavioral actions carried out by team members where team members draw from their individual resources (Burke et al., 2006b). Our study highlights the importance of experience as a clear advantage for the ability of teams to anticipate, monitor, and respond, and experience was equally valuable for all the teams. For teams in need of making decisions under a high degree of uncertainty, experience was highly important. For instance, in the coordinating teams, members used their experience when deciding which actions to take. They used their experience to anticipate what was likely to happen, and they were able to make decisions based on their deep knowledge of everyday work in the hospital (e.g., need for available beds, need for overbooking of beds, challenges due to regular staff being on leave, or specific changing weather conditions putting pressure on healthcare services), and the range of situations most likely to occur.

### 4.1. Relations, culture, and co-location

Figure 1 furthermore visualizes the varying importance of the factors that enable adaptive capacity within the four different types of teams studied. For instance, by being co-located, the structural and hybrid teams had the opportunity to develop what has been termed high quality relations (Havens et al., 2010), with frequent, timely, accurate, problem solving-communication. This not only enables the team to coordinate their work more effectively (Bolton et al., 2021), but also to develop shared goals, shared knowledge, and mutual respect within the team. These attributes increased the team’s ability to adapt to changes that occur (Gittell, 2008; Kozlowski et al., 2009), and the possibility to coordinate their work in response to adaptive triggers such as peak hours, acute alarms, and patient demands (Goldenhar et al., 2013; Grote et al., 2018). Team members prepared together and also looked out for each other and noticed when colleagues needed assistance without explicitly asking for help, all of which relates to the importance of relations and psychological safety in teamwork as described in the literature (Edmondson, 1999; Ceri-Booms et al., 2017; Bolton et al., 2021). Relational team factors stood out in our study as a significant enabler for adaptive capacity particularly for the structural and hybrid teams in both hospitals.

For the responsive and coordinating teams, however, it was challenging to develop these relations due to not working closely together on a regular basis. Instead, they compensated for this by having structural factors in place, like clear role descriptions and procedures that allowed them to function well as a team. Competent and experienced team members added to the likelihood of their success. In addition, the responsive teams regularly undertook simulation-based training focusing on communication to compensate for the lack of close relationships between the team members, as these teams only come together to perform specialized tasks for short periods of time.

In addition, the coordinating team in hospital 1 was heavily reliant on their software program to understand the total bed capacity situation, mainly due to the size of the team. The larger team in hospital 1 faced greater coordination challenges and had more difficulties developing and maintaining relations than smaller team in hospital 2 (Schmutz et al., 2019). The coordinating team in hospital 1 also relied more on having formal structures in place (e.g., meeting facilitator) compared to hospital 2, where the team was smaller, and the members more well acquainted with each other. This suggests, however, that the size of the team and perhaps the continuity of the team members are factors relevant to adaptive capacity. To a varying degree, both the responsive and the coordinating teams in the smaller hospital 2 had developed close relationships through frequent meetings. Although these teams relied strongly on set procedures in their day-to-day functioning, it was easier for them to decide on actions after initial disagreement due to the psychological safety their established relationships brought to the team (Edmondson, 2002; Schmutz et al., 2019). Our study indicates that no size fits all in terms of how to support these teams in promoting adaptive capacity and implies that team type and organizational settings need to be considered when developing teamwork improvements.

### 4.2. Structure (roles, organization of work, technology, and tools)

Figure 1 further shows that structuring factors such as roles, procedures, organization of work and provision of tools and technology are important factors for teams’ adaptive capacity. As everyday work in hospitals is characterized by constant fluctuations of work demands and changes to align with the situation, there must be room for maneuvering and flexibility. However, the flexibility has to be complemented by setting boundaries for a team’s degree of leeway (Burke et al., 2006a) to avoid the risk of maladaptation’s (Wears and Hettinger, 2014; Lyng et al., 2021). In our study we found that these boundaries are in many ways defined in the role descriptions and procedures of clinical work, which was fundamental for all teams, but especially in the coordinating and responsive teams only working together for short periods. Moreover, the set competence requirements, within the teams, safeguard the organizations from maladaptation’s due to unqualified personnel. Standardized procedures and formal task assignments can be conceptualized as stabilizing mechanisms. And, similar to the findings of Sanford (Sanford et al., 2022) and colleagues, we found that functional procedures and role descriptions, provide the team members with security in knowing how much, when, and how they can adapt. Therefore, the structural elements around the team are key for enabling adaptive capacity.

Previous research argues that aligning flexibility with stability is key for enabling adaptive capacity in teams (Grote et al., 2018; Salehi and Veitch, 2020). Relating this to our results, we found that for the structural and hybrid teams the respective hospital organizations had provided them with slack resources such as floating staff or staff with a coordinating role that provided the teams with the flexibility to adapt to different emerging situations (Saurin and Werle, 2017; Lyng et al., 2022), or adapt with the aim of maintaining the status quo on the ward, for instance changing the responsibility for work tasks to free up resources to handle deteriorating patients. Our results clearly showed that teams who were co-located and had developed sound relationships with each other (Gittell et al., 2010), could flex more effectively regarding roles and structure, especially to do with the allocation of tasks between team members. This improved the overall capacity of these types of teams, which would otherwise struggle when demands overloaded their capacity.

Overall, this study demonstrates that the adaptive capacity of all team types depended on the four main factors identified. However, the varying influence of the factors within the different teams, as depicted in Figure 1, indicates that different types of teams could need diverse types of structural parameters, training programs, leadership and relational emphasis when composing team in order to optimize everyday functionality and adaptive capacity. Further research should investigate both larger samples of teams, and how diverse organizational settings or national culture influences adaptive capacity in such types of healthcare teams.

### 4.3. Strengths and limitations

The study has some strengths and limitations. It is a major strength to combine observations and interviews in a total of eight teams in two different hospitals. This provided us with a rich material to understand adaptive capacity in teams and how team factor enables this. Although we have performed *in situ* observations of the teams, teamwork is dynamic and team members react to each other’s words and behaviors’ and to the demands of the environment (Anderson and Reedy, 2021). Adaptations most likely reflect these different factors. We did not observe all possible adaptations and their triggers, nonetheless, the results have been derived directly from empirical research using a theory driven combined deductive and inductive approach by a diverse team of researchers with clinical and academic expertise. By describing and aggregating the different factors into higher level themes, we have captured and defined the factors of importance for different types of hospital teams.

### 4.4. Implication/conclusion

This study has shown that factors for adaptive capacity in hospital teams must be seen in relation to the distinct attributes and circumstances of the teams in question. The key team factors that enable adaptive capacity are related to the technology and tools available for the teams; the specification of roles, procedures, and organization of work within the teams; the team members’ competence, and experience, and the internal culture in the team and the relationships between team members. We found that the influence of these factors on team adaptive capacity varied according to the team types. Adaptive capacity in structural and hybrid teams was mainly dependent on relational and cultural factors and by the team being co-located, as these teams had stable membership and roles. The responsive and coordinating teams, however, needed clear structures, roles, and tools and technology available to support their adaptive capacity, as the relational dimension was not as influential on task execution and adaptations within these teams as compared to structural and hybrid teams.

Our results imply that the systems supporting hospital teams must consider the teams’ strengths and weaknesses when organizing in and around the teams. Resilient responses to changes and challenges requires resources that are both relational and organizational (Gittell, 2008). The adaptive capacity of a team can be improved by joint reflections, team training and system improvement (Maynard et al., 2015; Schmutz et al., 2019). These results also indicate that hospitals could improve care quality by investing in competence, training, and activities to improve relations and collaboration in teams. The quality of teamwork is associated with the quality and safety of the care provided (Rosen et al., 2018), and sound team collaboration enhances the patient’s experience and outcome (Ashcroft et al., 2023). System improvement should focus on the internal structuring of teams such as ensuring continuity, procedure clarity, and available tools, etc., alongside the organization around teams and the division and coordination of the different complex tasks performed within and across teams in the organization. This could help prevent the types of adverse events and patient safety risks that often occurs during transitions of care (i.e., between care providers or during shift changes) (Rosen et al., 2018). The findings and implications from this study could likely be transferred to other parts of the healthcare system where the work is also carried out by teams. This includes, for example, the noted potential for improving teamwork by offering teams possibilities for reflection and training. However, team context and cultural traits need to be considered, as these are crucial for team operation and may differ significantly. Based on our study, we suggest further research should continue to explore factors for adaptive capacity in different team types, how a team’s attributes and circumstances affect its adaptability, and how the adaptive capacity of teams can be best supported.

## Data availability statement

The raw data supporting the conclusions of this article will be made available by the authors, without undue reservation.

## Ethics statement

The studies involving human participants were reviewed and approved by Regional committee for Medical and Health Research Ethics. The patients/participants provided their written informed consent to participate in this study.

## Author contributions

BF and HL conducted the data collection. BF conducted and transcribed all the interviews. BF, HL, VG, JA, and SW conducted the analysis and interpretation of data. All Authors developed the study design, contributed with writing, critical revision, and approval of the final version.

## Funding

This PHD project is part of the Resilience in Healthcare Research program which has received funding from the Research Council of Norway from the FRIPRO Toppforsk program, grant agreement no. 275367. The University of Stavanger, Norway, NTNU Gjøvik, Norway supports the study with kind funding.

## Conflict of interest

SW was member of Frontiers editorial board special topic; Occupational Health and Organizational Culture within a Healthcare Setting: Challenges, Complexities, and Dynamics.

The remaining authors declare that the research was conducted in the absence of any commercial or financial relationships that could be construed as a potential conflict of interest.

## Publisher’s note

All claims expressed in this article are solely those of the authors and do not necessarily represent those of their affiliated organizations, or those of the publisher, the editors and the reviewers. Any product that may be evaluated in this article, or claim that may be made by its manufacturer, is not guaranteed or endorsed by the publisher.
